# Transoropharyngeal closed reduction for traumatic atlantoaxial dislocation: a novel technique for fast and precise reduction

**DOI:** 10.1007/s00264-023-05817-7

**Published:** 2023-05-11

**Authors:** Yanlong Zhong, Yu Xin, Xuqiang Liu, Xinmao Xiao, Fengfen Guo, Haoqun Yao

**Affiliations:** 1 The Orthopedic Hospital, The First Affiliated Hospital of Nanchang University, 1519 Dongyue Avenue, Nanchang, 330209 Jiangxi China; 2 Department of Orthopedics, The People’s Hospital of Yi Chun City, Yi Chun, Jiangxi China

**Keywords:** Traumatic atlantoaxial dislocation, Closed reduction, Clinical application

## Abstract

**Purpose:**

The aim of this study is to introduce a new technique for the rapid and accurate reduction of traumatic atlantoaxial dislocation (TAAD) and to investigate its radiological and clinical outcomes.

**Methods:**

The clinical outcomes of 18 patients who were diagnosed with acute TAAD and underwent rapid transoropharyngeal closed reduction in our hospital were retrospectively analyzed from January 2015 to December 2020. Following general anaesthesia, all patients were immediately treated with oropharyngeal reduction under somatosensory evoked potential monitoring. The Japanese Orthopedic Association score, neck disability index and visual analog scale score for neck pain were used to evaluate clinical efficacy. Atlantodental distance, posterior atlantodental interval, and the clivus-canal angle were used to assess reduction and spinal cord compression.

**Results:**

The mean follow-up time was 23.3 months, with a range of 13–38 months. No neurovascular injury occurred during the operations. For all patients, the closed reduction method through the oropharynx under general anaesthesia was successful, and the success rate of reduction was 100%. All patients recovered uneventfully with marked improvement in clinical outcomes and imaging parameters (*P* < 0.01). Two patients developed mild postoperative dysphagia. One patient developed postoperative fever and pulmonary infection.

**Conclusion:**

Rapid trans-oropharyngeal closed reduction can safely, effectively, and rapidly reduce acute TAAD. This method provides a new strategy for treatment of the condition.

## Introduction

TAAD refers to trauma-induced, anatomical and physiological changes caused by the rupture of the transverse ligament and odontoid fracture, which can lead to compression and injury of the medulla oblongata, the cervical medulla, and nerve roots [1–3]. The atlantoaxial spine is adjacent to the upper cervical spinal cord and medulla oblongata and can compress the spinal cord after dislocation, causing local pain, decreased muscle strength, numbness in the limbs, urinary and defecation dysfunction, and, in severe cases, respiratory dysfunction that may be life-threatening [1]. Thus, early and safe reduction is key to treating the condition. Current methods of resetting are primarily bedside continuous cranial traction or cranial traction resetting under anaesthesia. However, continuous cranial traction reduction has a number of drawbacks including immediate reduction not being possible, potential aggravation of the spinal cord injury, difficulty with patient cooperation, and difficulty in reduction occurring after traction [4]. To effectively solve these issues, we propose a rapid transoral reduction method based on the anatomical structure of the atlantoaxial spine.

In 2005, Weisskopf et al. [4] reported on the treatment of eight patients with rotational fixed atlantoaxial dislocation by method similar to the one we are proposing. All eight patients were satisfactorily repositioned without redislocation, and five had complete resolution of pain after reduction. Subsequently, in 2018, Dezsoe et al. [5] reported satisfactory results with transoral reduction of atlantoaxial rotational subluxation in children and adolescents. In these initial instances of transoral closed reduction, most of the cases were relatively simple rotational fixed dislocations or subluxations that required little reduction force [5]. Hence, while the feasibility of transoral closed reduction was verified, due to the lack of strong longitudinal traction and rotation force, it remains difficult and risky for complex atlantoaxial dislocations—especially in patients with life-threatening emergencies.

In this study, we retrospectively followed 18 patients with confirmed TAAD who were treated with a novel closed reduction technique through the oropharynx. Objective clinical outcomes and radiological parameters were evaluated for at least 13 months following surgery. We hypothesized that this technique would be both safe and effective for achieving rapid and precise reduction of the atlantoaxial joint in these patients.

## Material and methods

### Study population

A retrospective analysis was conducted on 18 patients diagnosed with acute TAAD and treated with rapid oropharyngeal closed reduction in our hospital from January 2015 to December 2020. There were 14 males and four females, aged from two to 80 years included in the study. Eight patients had high-risk, traumatic dislocations, five with an atlantoaxial locking ring without neurological symptoms, and five patients were unable to cooperate with traction. All had a clear history of trauma. The causes of injury included car accident (five cases), fall from height (six cases), fall (five cases), neck sprain (one case), and neck massage (one case) (Table 1).

**Table 1:** Survey of patient data

Patient No.	Gender	Age(year)	COI	Tracheotomy	PTT (day)	Treatment	ROR	Complication	Follow-up (month)
1	M	7	Neck massage	Yes	No	TCR + PFF(C1-C2)	Complete	No	24
2	M	22	Car accident	No	No	TCR + PFF(C1-C2)	Complete	No	25
3	M	2	Car accident	No	Yes/3d	TCR + Brace fixation	Complete	No	24
4	M	53	Falls from height	No	No	TCR + PFF(C1-C2)	Complete	Dysphagia	24
5	F	71	Car accident	Yes	Yes /20d	TCR + PFF(C1-C2)	Complete	No	18
6	M	80	Falls from height	Yes	Yes /1d	TCR + PFF(C1-C2)	Complete	Pneumonia	14
7	M	57	Car accident	Yes	No	TCR + PFF(C1-C3)	Complete	No	13
8	M	38	Neck sprain	Yes	No	TCR + PFF(C1-C2)	Complete	No	16
9	M	36	Fall	No	Yes /14d	TCR + PFF(C1-C2)	Complete	No	28
10	M	42	Falls from height	No	No	TCR + Brace fixation	Complete	No	32
11	M	71	Fall	No	Yes /1d	TCR + PFF(C1-C2)	Complete	No	18
12	M	47	Falls from height	No	No	TCR + PFF(C1-C2)	Complete	No	19
13	F	13	Falls from height	No	Yes /7d	TCR + Brace fixation	Complete	No	24
14	M	39	Falls from height	No	Yes /1d	TCR + PFF(C1-C2)	Complete	Dysphagia	34
15	M	42	Drunken fall	Yes	No	TCR + Cervical collar fixation	Complete	No	28
16	M	63	Fall	No	No	TCR + PFF(C1-C2)	Complete	No	23
17	F	3	Fall	Yes	No	TCR + PFF(C1-C2)	Complete	No	19
18	F	4	Car accident	Yes	Yes /7d	TCR + Atlantoaxial fixation	Complete	No	38

*TCR*, transoropharyngeal closed reduction; *PFF*, posterior fixed fusion; *M*, male; *F*, female; *COI*, cause of injury; *PTT*, preoperative traction time; *ROR*, reduction on radiography

The clinical manifestations of patients with TAAD varied considerably (Table 2). The most common presenting symptom was occiput/neck pain and restricted neck movement in nine patients (50%). Quadriparesis occurred in eight patients (44.4%), dysuria in eight (22.2%), and coma in four patients (22.2%). Four patients showed sudden paraplegia with unconsciousness and respiratory distress due to trauma and required emergency tracheal intubation. Furthermore, another four patients underwent continuous cranial traction and organotomy with ventilator-assisted ventilation in the ICU before surgery.

**Table 2:** Principal presenting clinical symptoms in 18 Patients

Symptom	Number of patients (%)
Occiput/neck pain	9(50%)
Restricted neck movement	9(50%)
Quadriparesis	8(44.4%)
Dysuria	8(44.4%)
Dyspnea	8(44.4%)
Coma	4(22.2%)
Paresthesia	3(16.7%)
Dizziness	2(11.1%)

### Clinical function and radiographic outcome assessment

Pre and postoperative evaluations of clinical function outcomes in each patient included the following assessments: the Japanese Orthopedic Association (JOA) score [6], neck disability index (NDI) [7], and the visual analog scale score for neck pain (VASSNP) [8]. The JOA and NDI scoring systems were used to evaluate neurological status and neck conditions both before and after surgery, respectively. The VASSNP was used to assess pain recovery.

As patients with TAAD often require urgent care, it is crucial to carefully assess the patient’s condition and perform imaging examinations with pre-fixation of the neck for protection. All patients were evaluated using pre and postoperative computed tomography (CT) radiological measurements. The atlantodental interval (ADI) was measured to evaluate the horizontal dislocation of C1 over C2. The posterior atlanto-dental interval (PADI) was measured to evaluate the space available for the spinal cord. The clivus-canal angle (CCA) was measured to assess the extent of ventral compression of the spinal cord and medulla.

### Rapid transoralpharyngeal closure and reduction procedure

**Fig. 1:**
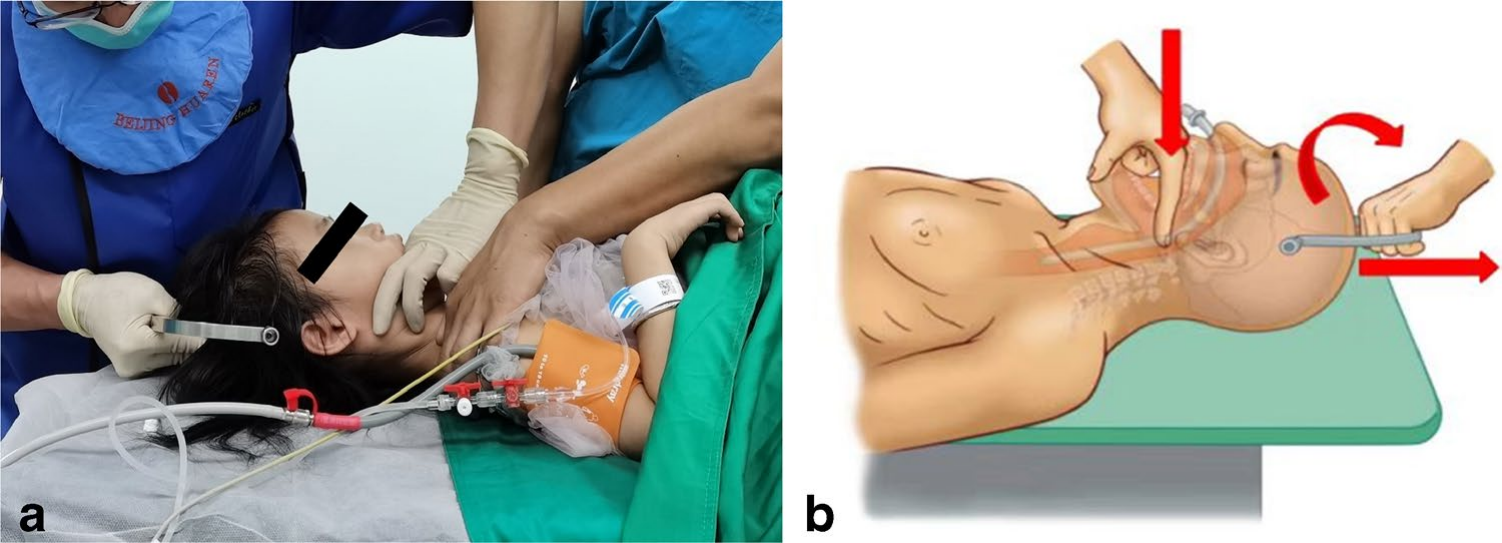
Schematic demonstrating use of reduction of atlantoaxial dislocation (AAD) by oropharyngeal manipulation (**a**). Three components of traumatic AAD resetting force: the longitudinal traction force of cranial traction, the resetting force of finger pressure, and the force to control the left and right rotation of the atlantoaxial spine through the traction arch plane (**b**)

The procedure for closed transoral reduction of TAAD (Fig. 1) is summarized as follows: (1) the patient is placed in the supine position with general anaesthesia via nasal intubation or basic anaesthesia only (tracheal intubation in high-risk patients may lead to aggravation of the spinal cord injury); (2) a semicircular cranial traction arch is placed, and both shoulders are fixed with adhesive tape or held by an assistant against traction. Under somatosensory evoked potential (SEP) monitoring, the cranial traction arch is tracted longitudinally with one hand, and one finger of the other hand touches the posterior pharyngeal wall through the oral cavity to sense the displaced atlas or cardinal vertebrae and then directly holds the dislocated anterior atlantoaxial arch or lateral block; (3) using the fingertip on the oropharynx as the fulcrum, the cranial traction arch is tracted longitudinally, and the cranial-atlantoaxial rotation plane is controlled using the traction arch to carefully rotate against the mechanism of injury; (4) if there is rotational dislocation of the atlantoaxial spine and locked facet, the cranial traction arch is longitudinally tracted based on reverse rotation to release the joint interlock. If the atlas was dislocated anteroposterior, the anterior edge of the anteriorly displaced atlas or axis was resisted to prevent the dislocation from aggravating. Successful reset can be directly felt and a popping sound is produced during joint reset. C-arm fluoroscopy can confirm the restoration of the normal anatomic sequence of the atlantoaxial positional relationship.

During the operation, general anesthesia is used for transnasal intubation, as transoral intubation may affect the operation. If the injury is serious, the risk of using intubation is high, or, if intubation is difficult for the patient and forced intubation is not recommended, then rapid reduction can be performed after basic anaesthesia and adequate muscle relaxation. Careful review of the preoperative films should be conducted, especially the preoperative 3D CT images, to understand the mechanism of injury and the direction of the atlantoaxial dislocation. On the basis of a clear mechanism of injury and direction of dislocation, the direction of the finger nudge reset and the traction arch rotation should reverse the injury, which is key to a successful reset. If there is an error in reading the preoperative film, incorrect positioning can occur during the operation and aggravate the dislocation and injury.

### Statistical analysis

Differences in pre and postoperative variables were tested by paired *t*-tests using IBM SPSS Statistics version 26.0 (IBM Corp., Armonk, New York, USA). A *P*-value < 0.05 was considered statistically significant.

## Results

In this study, 18 patients with TAAD had their injuries successfully repositioned by transoral pharyngeal closed reduction under general anaesthesia, and the reduction success rate was 100%. There were 13 cases of atlantoaxial fusion with posterior internal fixation, one case of non-fusion with simple fixation, three cases of external fixation with a simple brace, and 1 case of abandonment of treatment after reduction. The mean follow-up time was 23 months with a range of 13 to 38 months.

### Clinical outcomes

No adverse events such as neurovascular injury, postoperative wound infection, or internal fixation failure occurred. All of the patients exhibited improvement in postoperative nerve function compared to preoperatively. The preoperative JOA score was significantly improved compared to postoperative scores (15.4±1.0 vs. 10.4±1.9, *P* < 0.001). Furthermore, the NDI and VASNP scores were markedly decreased after surgery compared to preoperatively (*P* < 0.001) (Table 2). Two patients experienced mild dysphagia, which improved 6 months after surgery. One patient developed postoperative fever and pneumonia, but recovered after two weeks of antibiotics.

### Radiological outcomes

Radiographic fusion without loss of reduction was confirmed in all patients based on X-rays and CT scans at the final follow-up. We further analyzed pre and postoperative radiological measurements (Table 3). The postoperative mean anterior ADI was 2.4±0.8 mm, a 6.6±1.1 mm decrease compared with the preoperative mean (*P* < 0.001). Similarly, the SAC and CCA were significantly improved postoperatively (*P* < 0.001).

**Table 3:** Functional and radiological outcomes before and after surgery

Variable	Pre-operative	Post-operative	*P*
JOA score NDI (%)	10.4±1.9	15.4±1.0	< 0.001
	60.6±13.9	6.4±2.2	< 0.001
VASSNP	6.3±1.4	0.9±0.5	< 0.001
AADI (mm)	6.6±1.1	2.4±0.8	< 0.001
PADI (mm)	13.5±0.8	18.4±0.3	< 0.001
CCA(°)	128.2±7.9	144.1±4.8	< 0.001

*JOA*, Japanese orthopedic association; *NDI*, neck disability index; *VASSNP*, visual analog scale score for neck pain; *AADI*, anterior atlantodental interval; *PADI,* posterior atlantodental interval; *CCA*, clivus-canal angle.

NDI (%) of 10–28% is considered mild disability, 30–48% moderate, 50–68% severe, and ≥ 72% complete disability

### Illustrative cases

#### Patient 3

**Fig. 2:**
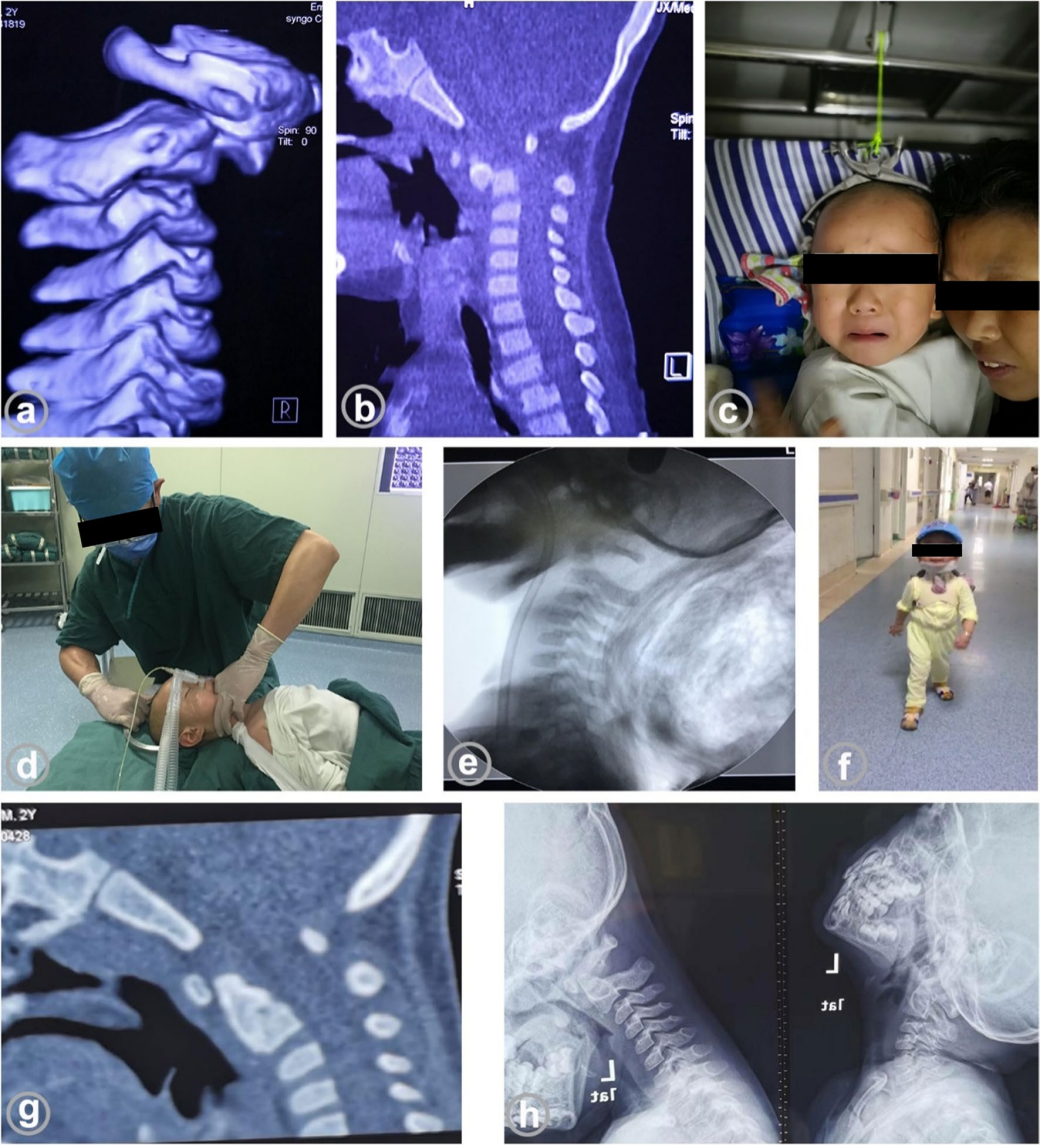
Pre-reduction computed tomography (CT) showing separation of the dentate epiphysis and atlantoaxial dislocation (**a**, **b**); the patient was unable to tolerate cranial traction (**c**); transoral closed reduction was performed under anesthesia (**d**); fluoroscopy confirmed successful manipulation (**e**); external fixation of head, neck and chest brace was performed and the patient walked normally (**f**); 6 months postoperative CT showed closure of the dentate epiphysis (**g**); 3 years postoperative cervical flexion and extension x-ray showing closure of the dentate epiphysis and no instability of the atlantoaxial spine (**h**)

A two year-old boy had a fall resulting in neck pain and limited motion. A CT examination ten days later revealed a separated dentate epiphysis and atlantoaxial dislocation (Fig. 2). The patient was unable to cooperate with cranial traction and was successfully repositioned via oropharyngeal closure after general anaesthesia, without surgical treatment. The child was fixed with a cephalothoracic brace for 12 weeks, and the epiphysis healed at three years of follow-up with no instability of the atlantoaxial spine.

#### Patient 5

**Fig. 3:**
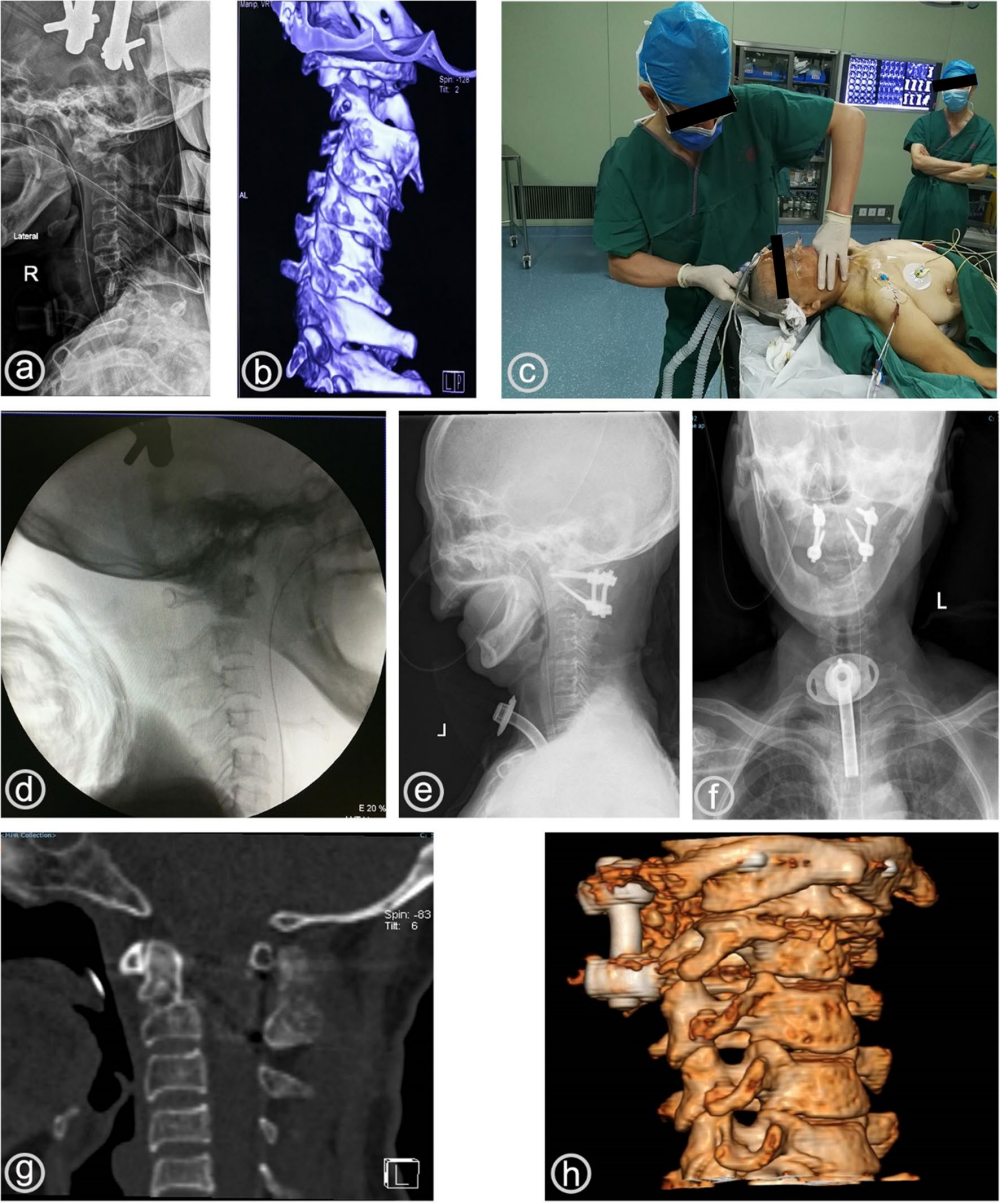
A bedside lateral radiograph 20 days after traction showing atlantoaxial subluxation and interlocking (**a**); 3D Computed tomography (CT) before reduction showing atlantoaxial subluxation with lateral block joint interlocking (**b**); closed reduction via the oropharynx after general anesthesia (**c**); intraoperative fluoroscopy after reduction showing release of atlantoaxial interlocking and correction of subluxation (**d**); postoperative review of anterior and lateral radiographs showing good internal fixation (**e**, **f**); CT sagittal view and 3D reconstruction showing atlantoaxial subluxation. The spinal cord compression was released and the atlantoaxial spine was fused with posterior implants (**g**, **h**)

A 71-year-old woman experienced tetraplegia and respiratory distress due to trauma from a car accident (Fig. 3). After hospital admission, a tracheotomy was performed in the intensive care unit and continuous cranial traction was given for 20 days; however, on review, the atlantoaxial subluxation remained uncorrected. An x-ray showed atlantoaxial subluxation and interlocking, and a CT scan further revealed lateral block interlocking. Under general anaesthesia, the patient underwent posterior atlantoaxial nail bar fixation and fusion after successful transoral closed reduction. The postoperative examination showed that the atlantoaxial subluxation was corrected and the spinal cord compression was released.

## Discussion

Most patients with traumatic atlantoaxial subluxation are in critical condition and should be treated quickly. In severe cases, such as those with spinal cord injury, vertebral artery occlusion, or the spread of oedema, the condition can be life-threatening [9].Therefore, the rapid and safe restoration of the physiological, anatomical sequence of the atlantoaxial spine and the mitigation of secondary neurological and vascular injuries in such cases are the primary issues to be addressed by clinicians [10].

**Fig. 4:**
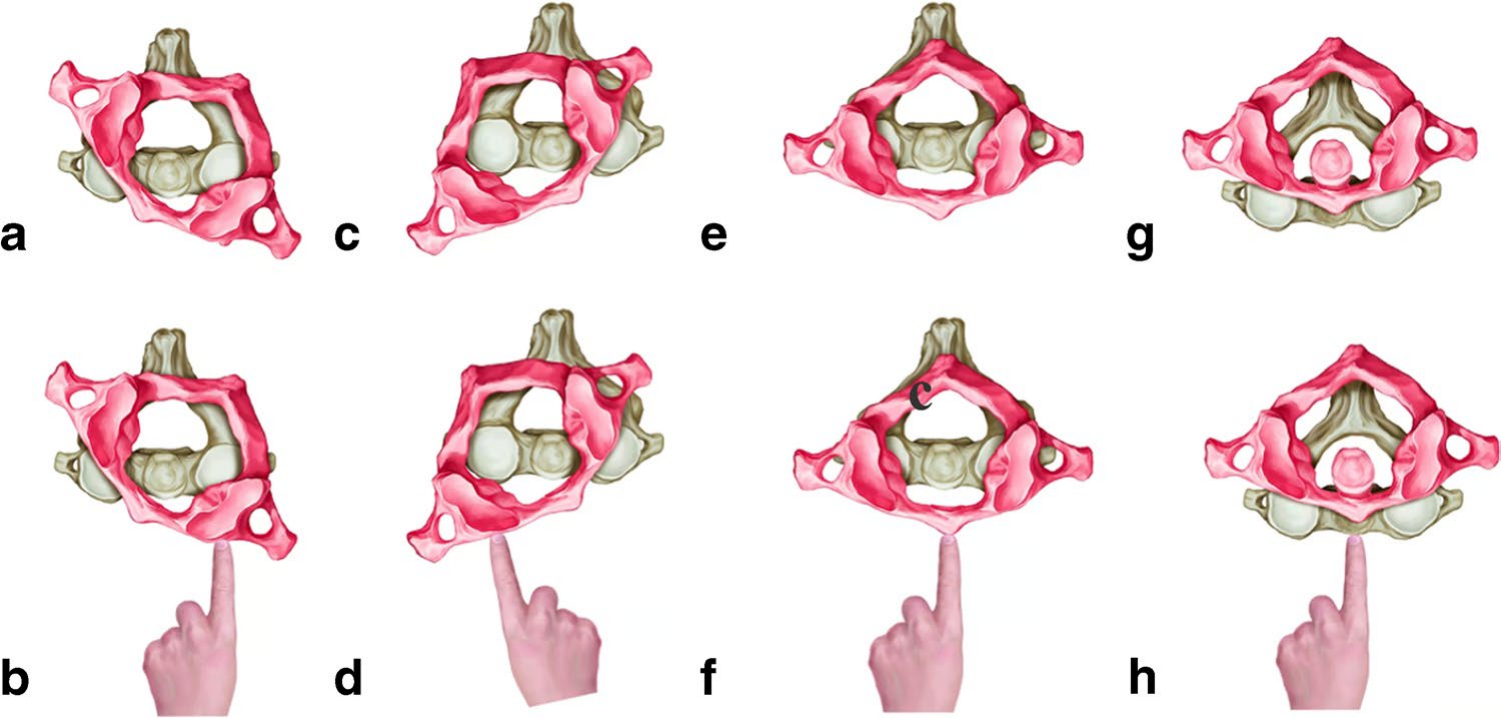
Left anterior rotational dislocation (**a**) via oropharyngeal reduction, with the top of the finger pressing the left anterior aspect of the atlas and pushing it backward (**b**); Right anterior rotational dislocation (**c**) via oropharyngeal reduction, with the finger top pressing the right anterior aspect of the atlas and pushing it backward (**d**); Anterior dislocation (**e**) via oropharyngeal reduction, with the fingers pressing against the anterior arch of the atlas and pushing back vertically (**f**); Posterior dislocation (**g**) via oropharyngeal reduction, with the top of the finger pressing the anterior edge of the axis vertebra and pushing vertically backward (**h**)

Currently, there are a number of TAAD subtypes, and the focus of each subtype is different. Fielding and Hawkings TAAD categories are divided into four subtypes based on the degree and direction of rotational subluxation and the presence of lateral block girdle fixation [11]. The Fielding subtypes are now accepted by most spine surgeons. However, based on the imaging data of our 18 TAAD cases, we propose a new typing approach based on the mechanism of TAAD, the direction of dislocation and the need for reduction (Fig. 4). In this approach, TAAD is divided into left anterior rotational dislocation, right anterior rotational dislocation, odontoid fracture with posterior dislocation and odontoid fracture (or no fracture) with anterior dislocation, and lateral displacement. Lateral displacement can rapidly change to one of the first four types with cranial traction. This typing approach allows for better visual representation of the relative displacement of atlantoaxial dislocations and helps to guide reduction.

The key to the treatment of TAAD is rapid, effective, and safe reduction. Previously, bedside cranial traction was performed while the patient was conscious [12, 13]. However, patients with acute TAAD can suffer from neck pain, mobility dysfunction, and neck muscle spasms, which can interfere with reduction. Continuous bedside occipo-mandibular banding or cranial traction can also cause chronic injury to the patient, such as occipital and cervical pain, body pressure sores, pulmonary and urinary tract infections, deep vein thrombosis, mental stress, and fear [14]. While TAAD is routinely repositioned by continuous cranial traction, the following types of cases are not appropriate for that method (but are a good indication for rapid transoral closed reduction): (1) when the atlantoaxial dislocation is life-threatening and there is a need for an immediate reset to lift compression off of the medulla oblongata or spinal cord; (2) when the atlantoaxial dislocation is combined with interlocking and sustained cranial traction reset is difficult and risky, and; (3) when special types and groups of fracture dislocation are not compatible with continuous traction such as in young children and in ankylosing spondylitis hunchback deformity patients.

Most patients with TAAD are in a critical state of high spinal cord compression, and traction may aggravate spinal cord injury. Furthermore, animal experiments have demonstrated that excessive traction may lead to spinal cord injury [15]. Hence, there is a need for close observation during cranial traction as potential damage to the spinal cord may occur with incorrect traction [4].

In our study, patients 9 and 11 were admitted without neurological symptoms, but were forced to abandon simple cranial traction after a single day due to obvious symptoms of neurological damage. This suggests that cranial traction alone is not a safe option for extremely unstable TAAD patients. As cranial traction is a purely longitudinal retraction force, for patients with anterior-posterior or left-right rotational interlocking of the lateral atlantoaxial block joints the lack of anterior-posterior or left-right rotational force makes the effectiveness of traction much less effective.

Weisskopf et al. [4] proposed a closed reduction technique in which one hand fixes the C2 spinous process through the body surface while the finger of the other hand presses the subluxation on the lateral block of the atlantoaxial spine through the posterior pharyngeal wall in a reverse rotation to reset the atlantoaxial subluxation. This technique has shown satisfactory results in children and adolescents with rotational subluxation of the atlantoaxial spine. This is a brave exploration of the transoral method of atlantoaxial dislocation, but it still has disadvantages that include (1) The body surface marker of the C2 spinous process is not obvious, and it is difficult to touch and fix the it, especially for patients with obesity or cervical dysplasia; (2) For cases of atlantoaxial joint locking, it is difficult to remove the locking because there is no longitudinal reduction force or excessive reduction through anterior compression, which is a risk for patients with an odontoid fracture, and; (3) As this technique primarily relies on finger pushing force, the reduction force is poor and is only suitable for acute, simple, adolescent rotational subluxation. Furthermore, the results are not sufficient for serious dislocations or long-term joint capsule contractures and the scope of adaptation is limited. In this study, we adopted the method of cranial traction with forces acting directly on the bone, which increased the longitudinal traction force of the atlantoaxial spine. Concurrently, we used the semi-annular cranial traction arch to control the cranial-atlantoaxial plane of rotation, which is an effective method for three-dimensional reduction of atlantoaxial subluxation. The longitudinal force of traction, the rotational force of the cranial-atlantoaxial plane, and the direct pressure of the index finger were the three important forces of reduction. Of these, the pressor pressure of the index finger on the atlas or axis is directly applied to the atlantoaxial region to add a longitudinal separating force. At the same time, the use of a semi-annular traction arch to control the rotational plane of the atlantoaxial spine worked to reduce the oropharyngeal index finger compression force and ensure the safety of the reduction.

Preoperative determination of the direction of atlantoaxial subluxation by 3D CT is critical. Since many patients with high-risk atlantoaxial subluxation have already undergone tracheal intubation or tracheotomy, it is not possible or necessary to include a magnetic resonance imagining examination. However, before resetting, a 3D CT examination of the atlantoaxial spine should be performed, and the direction of dislocation should be carefully analyzed to formulate a resetting plan.

Using the sense of finger’s touch is an important aspect of this operation during the reduction process. As the atlantoaxial bone is separated from the posterior pharyngeal wall by a thin layer of soft tissue, the index finger can easily reach the forward displaced atlas or pivot and apply pressure to reset the spine. When the reduction is successful, it is—through touch—easy to understand that the atlantoaxial plane has returned to a normal position. We recommend that oropharyngeal compression and cranial traction operations be performed by one surgeon, while an assistant pulls the patient’s shoulders for antagonistic traction. If available, the implementation of SEP monitoring during the reduction process can also help to increase the safety of procedure. In our study, was conducted during the reduction process in 12 cases, and no exacerbation of injury was observed.

The need for internal fixation after restoration can be comprehensively determined based on the damage to the stabilizing structures of the atlantoaxial spine. If there is clear evidence of transverse ligament injury or dentate fracture, atlantoaxial fixation and fusion can be performed directly after reduction, especially for high-risk patients. For cases with simple atlantoaxial interlock (possibly with anterior collapsed fractures of the C2 lateral mass) where the transverse ligament and dentate process are intact, cephalocervical-thoracic bracing can be used after reduction for protection. For atlantoaxial dislocation caused by C2 epiphyseal separation in children, if the dislocation has been ongoing over the short term and external fixation can maintain the reset, internal fixation should not be used. If the dislocation has been ongoing over the long term and there is contracture of the joint capsule, it is difficult to maintain the reset with external fixation. In these cases, temporary internal fixation can be used to maintain the reset, and the internal fixation can be removed after the epiphysis heals to restore functioning of the atlantoaxial spine.

Our method can also be applied to children with rotational ‘fixed’ atlantoaxial subluxation, which is mostly due to minor trauma or following an upper respiratory tract infection. The traditional treatment is occipomandibular or cranial traction. If transoral closed reduction is adopted, it can quickly correct the rotational subluxation state, reduce the pain of long-term traction in children, and reduce hospitalization times. Since atlantoaxial rotational subluxation in children requires less reduction force and is easier to reset, it also has wider clinical applications.

The study has several limitations that should be noted. First, it was a retrospective, single-centre study. Second, in contrast to transoropharyngeal closed reduction, there is a lack of another group evaluating clinical outcomes in patients undergoing other treatments. Third, the sample size is small. Therefore, future studies require larger prospective studies or randomized controlled trials with sufficient statistical power to further validate our results.

Rapid, transoral closed reduction as a tool in the treatment of acute TAAD is quick, safe, effective, and can provide a novel reduction method that avoids patient pain and risks associated with prolonged cranial traction.

## References

[1] Kepler CK, Vaccaro AR, Dibra F, Anderson DG, Rihn JA, Hilibrand AS, Harrop JS, Albert TJ, Radcliff KE (2014). Neurologic injury because of trauma after Type II odontoid nonunion. J Spine.

[2] Joaquim AF, Patel AA (2015). Surgical treatment of Type II odontoid fractures: anterior odontoid screw fixation or posterior cervical instrumented fusion?. Neurosurg Focus.

[3] Schroeder GD, Kepler CK, Kurd MF, Paul JT, Rubenstein RN, Harrop JS, Brodke DS, Chapman JR, Vaccaro AR (2015) A systematic review of the treatment of geriatric type II odontoid fractures. Neurosurgery Suppl 4:S6–S14 10.1227/NEU.0000000000000942 26378359

[4] Weisskopf M, Naeve D, Ruf M, Harms J, Jeszenszky D (2005). Therapeutic options and results following fixed atlantoaxial rotatory dislocations. Eur Spine J.

[5] Dezsoe J, Tamas F, Frank K, Daniel H, Markus L (2017). Transoral closed reduction of fixed Atlanto-Axial Rotatory-Subluxation (AARS) in childhood and adolescence. Clin Spine Surg.

[6] Association JO (1994). Scoring system for cervical myelopathy. Nippon Seikeigeka Gakkai zasshi.

[7] Vernon HT, Mior SA (1991). The Neck Disability Index: a study of reliability and validity. J Manipulative Physiol Ther.

[8] Huskisson EC (1974). Measurement of pain and pain threshold in patients with arthritis.

[9] Reddy K, Rao G, De Vi BI, Prasad P, Ramesh VJ (2009). Pulmonary function after surgery for congenital atlantoaxial dislocation: a comparison with surgery for compressive cervical myelopathy and craniotomy. J Neurosurg Anesthesiol.

[10] Noller C, Groah S, Nash M (2017). Inflammatory stress effects on health and function after spinal cord injury. Topics in spinal cord injury rehabilitation.

[11] Fielding J, Hawkins RJ (1977). Atlanto axial rotary fixation. (Fixed rotatory subluxation of the atlanto axial joint). J Bone Jt Surg.

[12] Jain VK (2004). Atlantoaxial dislocation. Neurol India.

[13] Spoor AB, Diekerhof CH, Bonnet M, Öner FC (2008). Traumatic complex dislocation of the atlanto-axial joint with odontoid and C2 superior articular facet fracture. Spine.

[14] Akbay A, Bilginer B, Akalan N (2014). Closed manual reduction maneuver of atlantoaxial rotatory dislocation in pediatric age. Childs Nerv Syst.

[15] Fujita Y, Yamamoto H (1989) An experimental study on spinal cord traction effect. Spine (Phila Pa 1976) 14:698–705. 10.1097/00007632-198907000-00009 10.1097/00007632-198907000-00009 2772718

